# Post-market outcome of an extract of traditional Cretan herbs on upper respiratory tract infections: a pragmatic, prospective observational study

**DOI:** 10.1186/s12906-017-1978-7

**Published:** 2017-09-21

**Authors:** Marilena Anastasaki, Antonios Bertsias, Stergios A. Pirintsos, Elias Castanas, Christos Lionis

**Affiliations:** 1 Clinic of Social and Family Medicine, School of Medicine, University of Crete, P.O. Box 2208, 71003 Heraklion, Greece; 2 Department of Biology, School of Sciences and Technology, University of Crete, 71003 Heraklion, Greece; 3 Botanical Garden, University of Crete, 74100 Rethymnon, Greece; 4 Laboratory of Experimental Endocrinology, School of Medicine, University of Crete, 71003 Heraklion, Greece

**Keywords:** Aromatic plants, Herbal extract, Upper respiratory tract infection

## Abstract

**Background:**

The beneficial effects of traditional herbs of Crete, Greece for the treatment of upper respiratory tract infections have been reported in observational and laboratory studies. Following a published, double blind, randomized, placebo controlled trial, this study aimed to assess the effectiveness of an extract of three Cretan herbs on the treatment of upper respiratory tract infections, upon its market release.

**Methods:**

An observational study was conducted in Heraklion, Crete, Greece. Participants were patients presenting at selected pharmacies with symptoms of upper respiratory tract infection, choosing to receive the extract for their treatment. Patients’ symptoms (local, general, total) where recorded at three time points within 1 week, using a questionnaire developed based on the Wisconsin Upper Respiratory System Survey. For each patient, symptoms were scored on a 0–7 Likert scale and three indexes were calculated: the score of local symptoms, the score of general symptoms and the total score of symptoms. Effectiveness was assessed by examining the reduction in these indexes over the 1-week observation period.

**Results:**

Mean score of general symptoms was 19.1 (SE: 0.9) in day 1, dropping to 8.6 (SE: 0.6) and 3.1 (SE: 0.4) in days 4 and 7 respectively. Mean score of local symptoms declined from 7.9 (SE: 0.5) in day 1 to 2.3 (SE: 0.3) in day 4 and to 0.5 (SE: 0.1) in day 7. Total score of symptoms reached 27.0 (SE: 1.2) in day 1, decreasing to 10.9 (SE: 0.8) in day 4 and to 3.5 (SE: 0.5) in day 7. The percentage of participants reporting fever was 82.1% at baseline, 8.0% in day 4 and 2.0% in day 7 (*p* < 0.0001 for paired differences). Multiple regression models indicated that supplementary medication intake did not seem to affect symptoms’ severity or the day patients reported that their symptoms ceased completely.

**Conclusions:**

This pragmatic study added evidence about the potential therapeutic effects of an extract of Cretan herbs on the amelioration of upper respiratory tract infection symptoms.

**Electronic supplementary material:**

The online version of this article (10.1186/s12906-017-1978-7) contains supplementary material, which is available to authorized users.

## Background

Aromatic plants have been historically used as remedies in the island of Crete, Greece [1]. Anthropological and laboratory research has explored their antioxidant activity and their effects on the alleviation of common cold symptoms [2, 3]. Such evidence led to the conduction of a double blind, randomized, placebo controlled trial aiming to test the effectiveness of an extract of three Cretan herbs, namely thyme (*Coridothymus capitatus* (L.) Rchb. f. synonym of *Thymbra capitata* (L.) Cav.), dictamnus (*Origanum dictamnus* L.) and sage (*Salvia fruticosa* Mill., *Salvia pomifera* L.), on the treatment of upper respiratory tract infections. The results indicated amelioration in the severity of symptoms, with 90% of treated patients being symptom-free at the last day of observation [4]. The extract was released in the market in 2015, in the form of nutritional supplement (extracts at a dilution of 15 ml/L in extra virgin olive oil, formulated as 0.5 ml soft-gel capsules, for a daily dose of two capsules).

The aim of this observational study was to explore the extract’s effectiveness on the treatment of upper respiratory tract infections, after its market release, through a questionnaire-based inquiry of participants. The primary objective was to assess the severity and duration of symptoms upon treatment. Secondary objectives included the investigation of the extract’s effectiveness on the presence of fever and in cases of supplementary medication intake.

## Methods

### Study design

An observational study was conducted in the prefecture of Heraklion, Crete, Greece (February – June 2016). Sixteen pharmacies, purposively selected, participated as recruiting sites. The preparation of the extract is extensively described elsewhere [4].

### Sample

Patients presenting at the pharmacies with symptoms of upper respiratory tract infection, who voluntarily requested and choose to purchase the extract as part of their treatment were recruited. Eligibility for inclusion in the study was based on the Jackson criteria for the identification of patients suffering from common cold [5]. In order also to increase the possibility that the infection was viral, and not of any other type, the presence of fever was considered as a perquisite for inclusion in the study. As such, inclusion criteria were: age over 18 years, sudden emergence of fever within 48 h from the visit in the pharmacy and presence of at least one of the symptoms: cough, sore throat, nasal discharge or ingestion, headache, muscle pain, sweating, rigors and fatigue. Exclusion criteria were: age under 18 years, absence of fever within 48 h from the visit to the pharmacy, daily consumption of aspirin (≥100 mg), presence of malignancies, immune system disorders, liver damage and pregnancy.

Setting as primary outcome the day patients reported that their symptoms ceased completely and assuming 5% probability of type-I error, a sample size of 185 patients had 90% power to detect an average time of symptoms’ cessation of 6 days, with standard deviation of ±1.2 days and accuracy of 0.17 days.

### Data collection

Upon presenting at the pharmacies, eligible patients were invited to complete an anonymous questionnaire (Additional file 1: Questionnaire.doc), developed based on the Wisconsin Upper Respiratory System Survey (WURSS-21), which assessed symptoms’ severity [6]. This tool has been previously used with reliable results [4]. Symptoms were recorded at the day of visit at the pharmacy (day 1), as well as at the fourth (day 4) and seventh (day 7) day of extract intake, via phone calls performed by the pharmacists.

Symptoms were classified as local (cough, sore throat, itchy throat, hoarseness, nasal congestion, excreta, sneezing and headache) and general (fatigue, muscle pain and rigors). They were scored in a Likert scale, with 0 denoting absence of symptom and 7 denoting maximal severity of the respective symptom. For each patient, three indexes were calculated: The score of local symptoms, the score of general symptoms and the total score of symptoms, defined as the sum of general and local symptoms’ scores. Presence of fever was recorded as a dichotomous variable (yes/no).

Collected socio-demographic and clinical characteristics included age (years), gender (male/female), BMI (Kg/m^2^), smoking (currently/former/never) and presence of chronic conditions (yes/no). Any supplementary medication received for the treatment of patients’ upper respiratory tract infection was also recorded (type and days of intake). The day patients reported feeling better and the day patients reported that their symptoms ceased completely were recorded as well.

### Statistical analysis

Sample characteristics were summarized using descriptive statistics. For comparisons between categorical variables Pearson’s χ^2^ tests were performed. Paired Samples T-test and McNemar tests were used for comparisons of paired differences. To assess the severity of all symptoms over the observation period, the Area under the Curve (AuC) was computed. Multiple linear regression models were performed with dependent variables: the AuC, the day patients reported feeling better and the day patients reported their symptoms ceased completely. Independent variables were the socio-demographic and clinical characteristics and supplementary medication intake (yes/no). Linear mixed models with dependent variables the total score of symptoms and presence of fever and independent variables the day of observation (1, 4, 7), supplementary medication intake (by day of observation), socio-demographic and clinical characteristics were used to assess symptoms’ alleviation over time. Confidence level was a = 0.05. Statistical software used were IBM SPSS version 21 and Stata SE version 12.

## Results

### Sample characteristics

A total of 493 patients with upper respiratory tract infection symptoms visited the selected pharmacies during recruitment. Among those, 315 (63.9%) met the inclusion criteria, 157 (50.2%) agreed to participate and 6 (3.8%) dropped-out. Data from 151 patients were analyzed (81.6% of the estimated target of 185 patients). Participants’ mean age was 43.6 (±17.6) years, while 95 (62.9%) were females. Mean BMI was 26.3 (±5.6) kg/m^2^, 68 (45%) participants were current smokers and 44 (29.1%) reported at least one chronic condition. A total of 85 (56.3%) participants received supplementary medication for the treatment of their upper respiratory tract infection at day 1. The respective percentage for the whole observation period was 64.9% (*n* = 98).

### Symptoms’ severity

**Fig. 1:**
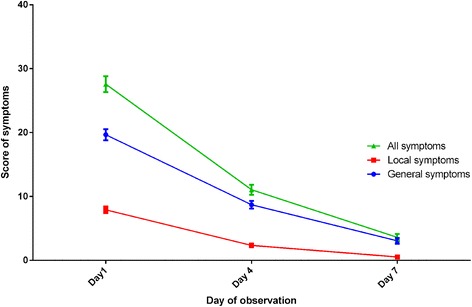
Mean symptoms’ scores and standard errors per day of observation

Table 1 presents the mean scores of general and local symptoms and the mean total score of symptoms during the observation period, along with the means of individual changes in symptoms’ scores between baseline and end of follow-up. Mean score of general symptoms was 19.1 (SE: 0.9) in day one, dropping to 8.6 (SE: 0.6) and 3.1 (SE: 0.4) in days 4 and 7 respectively, illustrating a decrease by 55.0% in day 4 and by 83.8% in day 7 (*p* < 0.001 for all paired differences). Mean score of local symptoms declined from 7.9 (SE: 0.5) in day 1 to 2.3 (SE: 0.3) in day 4 and to 0.5 (SE: 0.1) in day 7, showing a reduction by 70.9% in day 4 and by 93.7% in day 7 (*p* < 0.001 for all paired differences). Mean total score of symptoms reached 27.0 (SE: 1.2) in day 1, falling to 10.9 (SE: 0.8) in day 4 and to 3.5 (SE: 0.5) in day 7, indicating a decrease by 59.6% in day 4 and by 87.0% in day 7 (*p* < 0.001 for all paired differences; Fig. 1). On the first day, 124 (82.1%) patients reported having fever. This rate dropped to 8.0% at day 4 and to 2.0% at day 7 (*p* < 0.0001 for paired differences).

**Table 1:** Mean symptoms’ scores over the observation period

Symptoms	Mean	Standard error	Minimum	Maximum
General symptoms				
Day 1	19.1	0.9	1	45
Day 4	8.6	0.6	0	35
Day 7	3.1	0.4	0	35
Mean of individual change (day 1- day 7)	16.1	0.8	0	45
Local symptoms				
Day 1	7.9	0.5	0	21
Day 4	2.3	0.3	0	19
Day 7	0.5	0.1	0	14
Mean of individual change (day 1- day 7)	7.4	0.5	0	21
Total				
Day 1	27.0	1.2	2	66
Day 4	10.9	0.8	0	42
Day 7	3.5	0.5	0	35
Mean of individual change (day 1- day 7)	23.5	1.1	2	65

Patients reported feeling better on the third day of extract intake (median: 3.0, 95%CI: 3.0–3.5). They also reported that their symptoms had ceased completely by the sixth day of intake (median: 6.0, 95% CI: 5.0–6.0). By the end of the observation period 81.7% of our participants reported that their symptoms had ceased completely. The mean AuC for all symptoms was 26.2 (±17.4). Multiple linear regressions did not show any significant predictors (including supplementary medication intake) for the AuC, the day patients reported feeling better or the day patients reported that their symptoms ceased completely.

### Symptoms’ severity over time

Linear mixed models showed a significant decrease in the total score of symptoms over time (b = −14.6 in day 4 and b = −21.6 in day 7; *p* < 0.0001 for both) and the presence of fever (b = −0.8, *p* < 0.001 both for days 4 and 7). A borderline positive association between the total score of symptoms and supplementary medication intake was observed (b = 2.9; *p* = 0.065), indicating that patients with more severe symptoms tended to consume extra medication. Results indicated no significant interactions between supplementary medication intake and day of observation in the prediction of the total score of symptoms (b = −2.03; *p* = 0.362 for day 4 and b = −3.0; *p* = 0.306 for day 7). Post-hoc analyses indicated no significant differences in the total score of symptoms between patients consuming supplementary medication and those who did not (mean difference = 0.87; 95% CI: −2.5 - 4.26; *p* = 0.611 for day 4 and mean difference = −0.1; 95% CI: −5.1-5.0; *p* = 0.980 for day 7).

## Discussion

The present study follows a previously published work [4] on the effectiveness of a combination of Cretan herbs on the amelioration of upper respiratory tract infections. Indeed, our data indicated significant decrease in symptoms’ scores in days 4 and 7 of observation, compared to baseline. We documented a reduction of 90.3% in the percentage of people reporting fever between days 1 and 4 and 97.6% between days 1 and 7, a result that has not been reported previously. Supplementary medication intake did not seem to affect either the reduction on the severity of symptoms or the day patients reported that their symptoms ceased completely.

A literature review study reported 16 trials of herbal remedies for respiratory tract infections [7]. Trials have also examined the efficacy of *Salvia officinalis* products regarding certain respiratory tract infections, including acute sore throats [8] and acute pharyngitis [9], presenting positive effects in the treated groups. Our narrative search did not identify studies regarding the effectiveness of similar products upon their market release. However, we viewed our results in parallel to the results from the placebo group of a trial with similar population, as well as the placebo group of this extracts’ clinical trial. The first trial explored the efficacy of a *Pelargonium sidoides* preparation in common cold and reported that 11.8% of participants in the placebo group had complete clinical cure (according to cold intensity score) within 10 days of observation [10]. In the extract’s clinical trial, 70% of the participants in the placebo group had symptoms’ cessation by day 7 [4]. In the present study, 81.7% of the participants reported that their symptoms had ceased completely by day 7. Although results cannot be compared directly due to the differences in the design of the studies, this may provide an indication about the performance of the extract in relation to no actual treatment.

This study is prone to the limitations of its observational design, including the fact that there was no control group for comparable results. Additionally, information about the daily course of patients’ treatment could not be reported, since data was gathered at distinct time points. The presence of fever was, also, not recorded as exact temperature but rather as a dichotomous variable (yes/no). Almost two out of three patients received supplementary medication for their treatment. In all regression models, supplementary medication intake was modelled as dichotomous on each observation, since types and duration of intake differed for each patient. Thus, the effect of competitive active ingredients and types of medication could not be documented. Moreover, it was not possible to determine the extracts’ effectiveness on specific viral infections, since there was no identification of patients’ viral strains and viral loads. Finally, our sample was confined only to patients who voluntary requested the extract and were able to afford its purchase.

Despite these limitations, this study reflected the potential therapeutic effects of the extract, out of the controlled clinical trial setting, in the daily practice scenario where patients consume several medications at different time points for their treatment. In a period were debate regarding the cost-effectiveness of antiviral therapy is ongoing [11], further research on the use of aromatic plants may yield promising results.

## Conclusions

The findings of this study affirmed those of a previously published, double blind, randomized, placebo controlled trial, providing additional evidence about the potential therapeutic role of Cretan herbs in cases of upper respiratory tract infections.

## Additional file

Additional file 1: Questionnaire. (DOC 283 kb)

## Abbreviations

AuC: Area under the Curve
WURSS-21: Wisconsin Upper Respiratory System Survey
