# Supplementary material for: Post-market outcome of an extract of traditional Cretan herbs on upper respiratory tract infections: a pragmatic, prospective observational study
Source: BMC Complement Altern Med. 2017 Sep 21;17:466. doi: 10.1186/s12906-017-1978-7 (PMC5609057; doi:10.1186/s12906-017-1978-7)
Supplement: Additional file 1: — Questionnaire. (DOC 283 kb) [file 12906_2017_1978_MOESM1_ESM.doc]

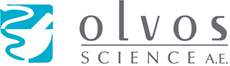


**INCIDENT RECORD FORM**

Post-market effectiveness of an extract of traditional Cretan herbs on upper respiratory tract infections

|  | | | | | | | |
| --- | --- | --- | --- | --- | --- | --- | --- |
| **Pharmacy code:** |  |  | **Number of patient:** |  |  |  |  |
| **Researcher**: (first name) □□□□□□□□□□□□  (last name) □□□□□□□□□□□□□□□□□□    **Date of first contact: __ / __ / 201_** | | | | | | | |

| ***ELIGIBILITY*** | | |
| --- | --- | --- |
| *Does the patient meet the eligibility criteria for inclusion?* | *□ YES* | *□ No* |
|  | | |
| ***If not the patient is not eligible for inclusion in the study*** | | |
| ***PATIENT’S INFORMED CONSENT*** | | |

|  |  | | | | | | | | | |
| --- | --- | --- | --- | --- | --- | --- | --- | --- | --- | --- |
| **Date of consent** (dd/mm/yyyy) : |  |  | / |  |  | / |  |  |  |  |

| **PATIENT DEMOGRAPHIC DATA** |
| --- |

| **date of birth** (dd/mm/yyyy) : |  |  | / |  |  | / |  |  |  |  |
| --- | --- | --- | --- | --- | --- | --- | --- | --- | --- | --- |

| **weight** : |  |  |  | , |  | kg | **height** : |  |  |  | cm |  |
| --- | --- | --- | --- | --- | --- | --- | --- | --- | --- | --- | --- | --- |

| **gender** : male: | □ | female: | □ |  |  |  |  |
| --- | --- | --- | --- | --- | --- | --- | --- |

| **SMOKING HISTORY** |
| --- |

🞎 **Non-smoker**

🞎 **Former smoker** *(Please, specify):

**When you used to smoke: Years of smoking**: □□ **Number of cigarettes per day**: □□

🞎 **Current smoker** * (Please, specify):

**Number of cigarettes per day**: □□ **Years of smoking**: □□

| **MEDICAL HISTORY** |
| --- |

Do you suffer from any chronic diseases?

| 🞎 No | 🞎 Yes | If yes, please specify : |
| --- | --- | --- |

| **Chronic disease/condition** | **Medication received** |
| --- | --- |
| 1. |  |
| 2. |  |
| 3. |  |
| 4. |  |
| 5. |  |
| 6. |  |
| 7. |  |

**Did you buy/receive any additional medication for the treatment of your respiratory infection?**

| 🞎 No | 🞎 Yes | If yes, please specify: |
| --- | --- | --- |

| **Medication name** | **Medication category (antibiotics, decongestants, etc.)** |
| --- | --- |
| 1. |  |
| 2. |  |
| 3. |  |
| 4. |  |
| 5. |  |
| 6. |  |

| **UPPER RESPIRATORY TRACT INFECTION SYMPTOMS – DAY 1** |
| --- |

| **Local symptoms** | **0**  **(No symptoms)** | **1**  **(Very mild)** | **2** | **3**  **(Mild)** | **4** | **5**  **(Moderate)** | **6** | **7**  **(Severe)** |
| --- | --- | --- | --- | --- | --- | --- | --- | --- |
| 1. Dry cough |  |  |  |  |  |  |  |  |
| 1. Sore throat |  |  |  |  |  |  |  |  |
| 1. Itchy throat |  |  |  |  |  |  |  |  |
| 1. Hoarseness |  |  |  |  |  |  |  |  |
| 1. Congested nose |  |  |  |  |  |  |  |  |
| 1. Nasal secretions |  |  |  |  |  |  |  |  |
| 1. Sneezing |  |  |  |  |  |  |  |  |
| 1. Headache |  |  |  |  |  |  |  |  |
|  | | | | | | | | |
| **General symptoms** |  | | | | | | | |
| 1. Fever   (Did you have fever today?) | **Yes / No** | | | | | | | |
|  | **0**  **(No symptoms)** | **1**  **(Very mild)** | **2** | **3**  **(Mild)** | **4** | **5**  **(Moderate)** | **6** | **7**  **(Severe)** |
| 1. Weakness/fatigue |  |  |  |  |  |  |  |  |
| 1. Muscle/joints’ pain |  |  |  |  |  |  |  |  |
| 1. Shivers |  |  |  |  |  |  |  |  |

**From which source were you informed about the extract?** (Please, record all that apply)

| 🞎 TV | 🞎 Radio | 🞎 Internet | 🞎 Newspaper | | 🞎 Friend/relative |
| --- | --- | --- | --- | --- | --- |
| 🞎 My pharmacist | | 🞎 My doctor | | 🞎 Other _________________ | |

**What were the reason/reasons that you chose the extract?**

(Please, specify all that apply)

| 🞎 Read/heard positive comments regarding its effectiveness |
| --- |
| 🞎 My doctor recommended as part of my treatment  🞎 My pharmacist recommended as part of my treatment |
| 🞎 A friend/relative recommended it as part of my treatment |
| 🞎 I want to use herbal medical products as part of my treatment |
| 🞎 I want to try it and ascertain its effectiveness |
| 🞎 Other reason _________________________________________________________________________ |

| **UPPER RESPIRATORY TRACT INFECTION SYMPTOMS – DAY 4** |
| --- |

| **Local symptoms** | **0**  **(No symptoms)** | **1**  **(Very mild)** | **2** | **3**  **(Mild)** | **4** | **5**  **(Moderate)** | **6** | **7**  **(Severe)** |
| --- | --- | --- | --- | --- | --- | --- | --- | --- |
| 1. Dry cough |  |  |  |  |  |  |  |  |
| 1. Sore throat |  |  |  |  |  |  |  |  |
| 1. Itchy throat |  |  |  |  |  |  |  |  |
| 1. Hoarseness |  |  |  |  |  |  |  |  |
| 1. Congested nose |  |  |  |  |  |  |  |  |
| 1. Nasal secretions |  |  |  |  |  |  |  |  |
| 1. Sneezing |  |  |  |  |  |  |  |  |
| 1. Headache |  |  |  |  |  |  |  |  |
|  | | | | | | | | |
| **General symptoms** |  | | | | | | | |
| 1. Fever   (Did you have fever today?) | **Yes / No** | | | | | | | |
|  | **0**  **(No symptoms)** | **1**  **(Very mild)** | **2** | **3**  **(Mild)** | **4** | **5**  **(Moderate)** | **6** | **7**  **(Severe)** |
| 1. Weakness/fatigue |  |  |  |  |  |  |  |  |
| 1. Muscle/joints’ pain |  |  |  |  |  |  |  |  |
| 1. Shivers |  |  |  |  |  |  |  |  |

**Did you receive any additional medication/s for the treatment of your respiratory infection?**

| 🞎 No | 🞎 Yes | If yes, please specify : | |  |
| --- | --- | --- | --- | --- |
| **Medication name** | | | **Day – date (from - until)** | |
| 1. | | |  | |
| 2. | | |  | |
| 3. | | |  | |
| 4. | | |  | |
| 5. | | |  | |
| 6. | | |  | |

| **UPPER RESPIRATORY TRACT INFECTION SYMPTOMS - DAY 7** |
| --- |

| **Local symptoms** | | **0**  **(No symptoms)** | | **1**  **(Very mild)** | **2** | | | **3**  **(Mild)** | | **4** | | **5**  **(Moderate)** | **6** | | **7**  **(Severe)** | |
| --- | --- | --- | --- | --- | --- | --- | --- | --- | --- | --- | --- | --- | --- | --- | --- | --- |
| 1. Dry cough | |  | |  |  | | |  | |  | |  |  | |  | |
| 1. Sore throat | |  | |  |  | | |  | |  | |  |  | |  | |
| 1. Itchy throat | |  | |  |  | | |  | |  | |  |  | |  | |
| 1. Hoarseness | |  | |  |  | | |  | |  | |  |  | |  | |
| 1. Congested nose | |  | |  |  | | |  | |  | |  |  | |  | |
| 1. Nasal secretions | |  | |  |  | | |  | |  | |  |  | |  | |
| 1. Sneezing | |  | |  |  | | |  | |  | |  |  | |  | |
| 1. Headache | |  | |  |  | | |  | |  | |  |  | |  | |
|  | | | | | | | | | | | | | | | |  |
| **General symptoms** |  | | | | | | | | | | | | | | |  |
| 1. Fever   (Did you have fever today?) | **Yes / No** | | | | | | | | | | | | | | |  |
|  | **0**  **(No symptoms)** | | **1**  **(Very mild)** | | | **2** | **3**  **(Mild)** | | **4** | | **5**  **(Moderate)** | | | **6** | **7**  **(Severe)** |  |
| 1. Weakness/fatigue |  | |  | | |  |  | |  | |  | | |  |  |  |
| 1. Muscle/joints’ pain |  | |  | | |  |  | |  | |  | | |  |  |  |
| 1. Shivers |  | |  | | |  |  | |  | |  | | |  |  |  |

| **Description of illness:** | |
| --- | --- |
| How many days did you lose from work? |  |
| When did you start to feel better?  (Please, record the date) |  |
| When did you return to your normal daily activities?  ( Please, record the date ) |  |
| Which was the day of complete cessation of your symptoms? |  |

**Did you receive any additional medication/s for the treatment of your respiratory infection?**

| 🞎 No | 🞎 Yes | If yes, please specify : | |  |
| --- | --- | --- | --- | --- |
| **Medication name** | | | **Day – date (from - until)** | |
| 1. | | |  | |
| 2. | | |  | |
| 3. | | |  | |
| 4. | | |  | |
| 5. | | |  | |
| 6. | | |  | |

| STATEMENT OF PHRMACIST/RESEARCHER |
| --- |

I confirm that I approve all data recorded in this document and that the provided information accurately reflects the facts that took place during the patients’ participation in the study.

|  |  |  |
| --- | --- | --- |

Pharmacist’s/researcher’s signature Date
